# Characterizing Autophagy in the Cold Ischemic Injury of Small Bowel Grafts: Evidence from Rat Jejunum

**DOI:** 10.3390/metabo11060396

**Published:** 2021-06-17

**Authors:** Ibitamuno Caleb, Luca Erlitz, Vivien Telek, Mónika Vecsernyés, György Sétáló, Péter Hardi, Ildikó Takács, Gábor Jancsó, Tibor Nagy

**Affiliations:** 1 Institute of Surgical Research and Techniques, University of Pécs Medical School, 7624 Pécs, Hungary; lucaerlitz@gmail.com (L.E.); telek.vivien@pte.hu (V.T.); hardipet@gmail.com (P.H.); dr.takacsildi@gmail.com (I.T.); jancsogabor@hotmail.com (G.J.); ntibor85@gmail.com (T.N.); 2 Central Electron Microscope Laboratory, Institute of Medical Biology, University of Pécs Medical School, 7624 Pécs, Hungary; kali29@gmail.com (M.V.); gyorgy.setalo.jr@aok.pte.hu (G.S.J.)

**Keywords:** autophagy, apoptosis, ischemia–reperfusion, cold preservation, mucosal injury, small bowel grafts

## Abstract

Cold ischemic injury to the intestine during preservation remains an unresolved issue in transplantation medicine. Autophagy, a cytoplasmic protein degradation pathway, is essential for metabolic adaptation to starvation, hypoxia, and ischemia. It has been implicated in the cold ischemia (CI) of other transplantable organs. This study determines the changes in intestinal autophagy evoked by cold storage and explores the effects of autophagy on ischemic grafts. Cold preservation was simulated by placing the small intestines of Wistar rats in an IGL-1 (Institute George Lopez) solution at 4 °C for varying periods (3, 6, 9, and 12 h). The extent of graft preservation injury (mucosal and cellular injury) and changes in autophagy were measured after each CI time. Subsequently, we determined the differences in apoptosis and preservation injury after activating autophagy with rapamycin or inhibiting it with 3-methyladenine. The results revealed that ischemic injury and autophagy were induced by cold storage. Autophagy peaked at 3 h and subsequently declined. After 12 h of storage, autophagic expression was reduced significantly. Additionally, enhanced intestinal autophagy by rapamycin was associated with less tissue, cellular, and apoptotic damage during and after the 12-h long preservation. After reperfusion, grafts with enhanced autophagy still presented with less injury. Inhibiting autophagy exhibited the opposite trend. These findings demonstrate intestinal autophagy changes in cold preservation. Furthermore, enhanced autophagy was protective against cold ischemia–reperfusion damage of the small bowels.

## 1. Introduction

Intestinal transplant (Itx) is indicated for patients presenting life-threatening complications due to long-term total parenteral nutrition, short bowel syndrome, or intestinal failure [1,2]. Recent advancements in immunosuppressive pharmacology, surgical techniques, and postoperative care have contributed greatly to making this procedure a valid therapeutic option [2]. However, complications still exist with Itx, some of which are essentially caused by the unavoidable ischemia and the ensuing reperfusion of the organ [3]. Minimizing ischemic damage to the intestinal graft is, therefore, crucial. Thus, effective measures should be undertaken to ensure its viability [1]. Currently, the widespread strategy for preserving the intestine involves standard retrograde vascular perfusion followed by static cold storage (SCS) [4]. Although hypothermic storage is deployed to avert adverse intestinal insult, with time, grafts still deteriorate [3,5]. This is primarily due to the total absence of tissue perfusion and microcirculation during preservation. The developing tissue hypoxia will result in reduced adenosine triphosphate (ATP) formation, decreased glycolysis (shift to anaerobic type), increased acidosis, and dysfunction of energy-dependent enzymes [6]. The alterations present at the end of the storage period are worsened by reperfusion, initiating a complex injury cascade referred to as ischemia–reperfusion injury (IRI) [7]. Several parameters are used in addition to the mucosal changes to evaluate this ischemic damage of the intestine [8,9]. Ischemic injuries may result in graft dysfunction or non-function and chronic organ failure in the recipient. Advanced IRI of the intestinal graft is also followed by bacterial translocation, post-reperfusion syndrome, and massive fluid and electrolyte shifts, secondary to the impairment of its mucosal barrier [3]. Hence, the current clinical consensus on intestinal preservation allows only for a storage time of less than 10 h [3,10]. Within this limit, evidence suggests that the intestines will only exhibit moderate pathological changes [10].

Ischemic events deprive the affected cell populations of their oxygen and nutrient requirements, resulting in states of starvation and stress. To sustain core cellular function, these cells undergo a starvation-induced ‘’self-eating’’ process known as autophagy [11]. The autophagy pathway involves the formation of a membrane-bound vesicle, called the autophagosome, that encircles cytoplasmic proteins and organelles. Autophagosome formation is generally under the control of autophagy-related genes (Atg) and associated proteins [12]. Some of the well-studied Atg proteins include Beclin-1 and the microtubule-associated protein 1 light chain 3 (LC3) proteins. By interacting with the autophagy-specific class III PI3k complex, Beclin-1 can initiate autophagy. In contrast, modification of the LC3 protein to LC3II is considered an essential step for the maturation of autophagosomes [13,14,15]. Both Beclin-1 and LC3II proteins are frequently used to monitor the autophagy process [15,16]. The mature autophagosomes will fuse and empty their contents into lysosomes for degradation. The degradation products are then released back into the cytoplasm and re-utilized to aid metabolism [12,13]. Studies into autophagy using various models show that this pathway is rapidly upregulated under conditions of starvation, hypoxia, or ischemia [14,17,18,19,20,21].

Extended periods of ischemia can also elicit an apoptotic response in cells. Apoptosis is often described as the major form of cell death during warm and cold ischemia [22]. Increased caspase-3 activation and enterocyte apoptosis after varying periods of cold preservation have also been depicted in intestinal models [6,23]. Apoptosis and autophagy have a complex relationship [24,25]. Typically, an early autophagic response is associated with cell survival. In contrast, the upregulation of apoptosis will hasten cell demise, in some cases, by even inactivating autophagy [24,25]. The interaction between both pathways has been described as important for organ survival during ischemia [19,26,27,28]. 

The role of autophagy during cold preservation tends to show variation amongst the different organs. In the kidney, for example, a protective effect of autophagy during cold preservation has been described in the literature and that inhibition of this pathway accelerates ischemic injury [26]. Meanwhile, in the lungs, the opposite has been suggested [17,20]. Therefore, understanding the influence of autophagy on intestinal preservation may prove significant. Previous studies on the intestine have shown that disruption of the epithelial barrier by amino acid deprivation can induce protective autophagy in intestinal epithelial cells [29]. More so, the benefits of regulating autophagy have been demonstrated in the warm IRI of the intestine [14]. Hence, this study is designed to explore the changes in autophagy during the cold preservation of small bowel grafts. Furthermore, we determine whether modulating this pathway can play a protective role in the intestinal ischemia induced by cold storage. 

## 2. Results

### 2.1. Preservation Injury of Small Bowel Grafts

#### 2.1.1. Histology

We evaluated the extent of intestinal histological injury amongst the groups according to Park/Chiu’s classification system [8]. Intestinal mucosal injury was significantly higher in all the preservation groups (3, 6, 9, and 12 h) compared to the operated control group (0 h) (*p* ≤ 0.0001). High injury grades (Park/Chiu ≥ 4), characterized by frequently denuded villi, loss of villous tissue, and injured crypts, occurred more frequently after 12 h of preservation (4.27 ± 0.10) (Table 1).

**Figure 1:**
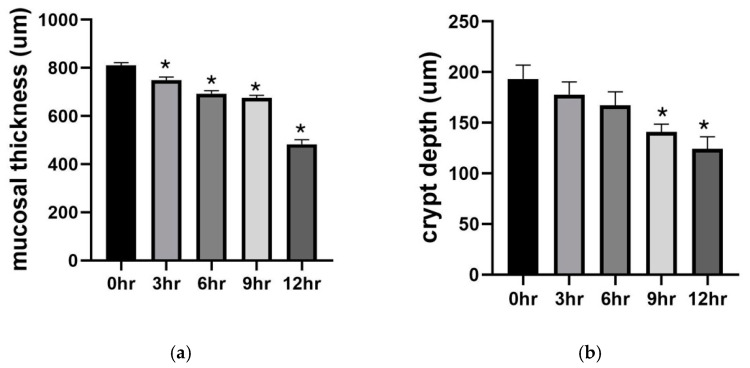
Morphometric analysis of the mucosa. Panel (**a**) shows changes in mucosal thickness. Panel (**b**) shows changes in crypt depth. Data are mean ± SEM, n = 6. * *p* < 0.05 versus the 0 h group (operated control).

By quantitative analysis, we also measured changes in mucosal thickness and crypt depth as indicators of mucosal atrophy. A significant decrease in mucosal thickness in the 3, 6, 9, and 12 h preservation groups was observed when compared to the operated control (*p* < 0.005) (Figure 1a). Similarly, crypt depth decreased with increasing time. This decrease was significant after 9 and 12 h of preservation (*p* = 0.0103 and *p* = 0.0005, respectively) (Figure 1b).

#### 2.1.2. Goblet Cell Abundance

**Figure 2:**
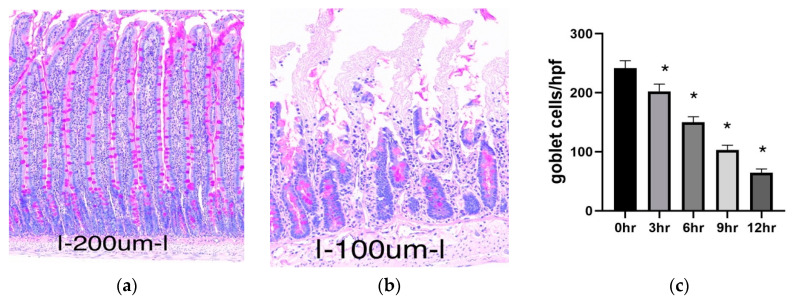
Goblet cell abundance in intestinal mucosa. Panel (**a**) is a representative photograph taken from the operated control (0 h) group. Panel (**b**) is a representative photograph taken from the 12 h group. The goblet cells containing mucin are stained purple (PAS staining positive). Panel (**c**) represents the changes in goblet cell numbers among the groups. Data are mean ± SEM, n = 6. * *p* < 0.05 increase versus the 0 h group (operated control).

Goblet cells are present in the entire intestinal mucosa and secrete mucin, which is protective against luminal insults and bacteria gaining access to the epithelium. They are rapidly depleted in response to ischemia, whereas they contribute significantly to mucosal restitution after ischemia–reperfusion [30]. Using periodic acidic Schiff (PAS) staining, we were able to estimate the presence of mucin-containing goblet cells in the mucosa of our experimental groups. From the results, the number of goblet cells decreased slightly but significantly in the 3 h group compared to the operated control (*p* = 0.0254). The decrease was more pronounced in the 6, 9, and 12 h groups (*p* < 0.0001) (Figure 2).

#### 2.1.3. Biochemical Analysis 

By biochemical analysis of the preservation fluid, we quantified the released lactate dehydrogenase enzyme (LDH) and lactate from the preserved grafts. LDH, as a general marker of cell injury, has been associated with ischemic events in the intestine. Similarly, changes in lactate are frequently used as a marker of cellular hypoxia [9,31]. The results show a significantly increased concentration of both markers in the 6 h group and the 12 h group in contrast to the 0 h group (LDH: *p* < 0.0001; lactate: *p* < 0.0001) (Table 2).

### 2.2. Autophagy Changes during Cold Preservation of Small Bowel Grafts

**Figure 3:**
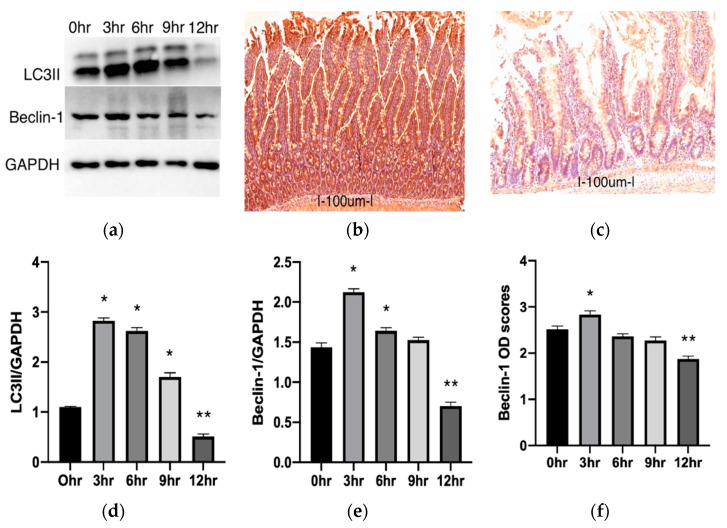
Changes of autophagy in intestinal mucosa. Panel (**a**) shows representative electrophoresis patterns for LC3II and Beclin-1. Panels (**b**,**c**) are representative of immunostaining for Beclin-1 at 0 h (**b**) and 12 h (**c**). Scale bar: 100 μm; Mg: 14.0x. Panels (**d**,**e**) show changes in LC3II and Beclin-1, respectively, with time. Each protein is represented as a ratio of GAPDH. Panel (**f**) illustrates changes in optical density (OD) scores for Beclin-1 immunostaining. Data are mean ± SEM, n = 6. * *p* < 0.05 increase versus the 0 h group (operated control). ** *p* < 0.05 decrease versus the 0 h group.

To quantify autophagic changes in intestinal tissues following cold preservation, the protein expression of two common markers of autophagy, LC3II and Beclin-1, were examined using immunoblot and immunostaining techniques. Our Western blot results for LC3II illustrated a significant increase in the 3, 6, and 9 h groups compared to the control (*p* < 0.0001) (Figure 3a,d). In the 12 h group, the LC3II protein expression was significantly decreased (*p* < 0.0001). Results for the Western blot analysis of Beclin-1 also showed a significant increase in the 3 and 6 h groups than in the control (*p* < 0.0001 and *p* = 0.0052, respectively). After 9 h, the values were higher than the control but not to the level of significance (*p* = 0.4007) (Figure 3a,e). In the 12 h group, a significant decrease was also observed compared to the control (*p* < 0.0001). A similar trend was noted in the immunohistochemical staining for the Beclin-1 protein. This method stains the cytosolic presence of Beclin-1. A darker stain signifies more prominence of the protein, and this can be quantified by measuring optical density (OD). Compared to the control, average OD scores were significantly increased in the 3 h group and significantly decreased in the 12 h group (*p* = 0.0095 and *p* < 0.0001, respectively) (Figure 3b,c,f).

We also observed the presence of autophagosomes in the cytoplasm of intestinal epithelial cells (those that have microvilli) using electron microscopy. When compared, more autophagosomes could be observed in the 6 h group than the 0 h or 12 h group. Representative electron microscopy photographs are available as Supplementary Material (S1).

### 2.3. Effects of Regulating Autophagy during Cold Preservation of Small Bowel Grafts

#### 2.3.1. Changes in Autophagic Activity

Significant mucosal disintegration and autophagy decline were observed after 12 h of storage in our first study. Therefore, we adopted this extended preservation duration for our second experiment series. To access the influence of autophagy, we treated separate groups with rapamycin (Rapa) or 3-methyladenine (3-MA) shortly before organ retrieval. Rapamycin is frequently used for promoting autophagy, while 3-MA is a known inhibitor of starvation-induced autophagy [14,15]. An untreated group served as a cold preservation control (PC). We verified the influence of these pharmacological agents on autophagic activity by performing immunostaining for Beclin-1 and p62/sqstmi proteins at the beginning (0 h), during (6 h), and at the end of cold preservation (12 h). p62/sqstmi (simply, p62) is another widely used autophagy marker that is selectively incorporated into the autophagosome and degraded by autophagy. The level of p62 protein inversely correlates with autophagic activity [14]. Additionally, at the end of preservation, we performed immunoblot staining for LC3II and Beclin-1 proteins.

**Figure 4:**
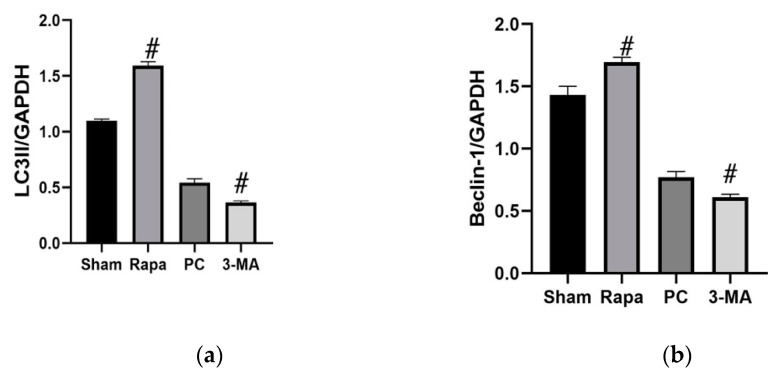
Changes in autophagy after 12 h long preservation. Panels (**a**,**b**) show changes in immunoblot for LC3II and Beclin-1, respectively. Each protein is represented as a ratio of GAPDH. Data are mean ± SEM, n = 6. # *p* ≤ 0.05 versus PC. Electrophoresis patterns for LC3II and Beclin-1 are available as supplementary materials (S2.2).

Immunostaining for Beclin-1 and p62 showed no significant difference amongst the groups after organ retrieval. By the 6 h mark, Beclin-1 OD scores for the Rapa group were significantly higher (*p* = 0.0072), while those for 3-MA group were significantly less (*p* = 0.0337) than for the PC group. At the end of the preservation period, immunostaining for Beclin-1 in the Rapa-group was still significantly more intense than the control (*p* < 0.0001). The 3-MA group scores were lower but not to a level of statistical significance (*p* = 0.48400). Immunostaining for p62 in the Rapa group had significantly lower OD scores than the PC group after both 6 and 12 h of preservation (*p* = 0.0318 and *p* = 0.0007, respectively). In contrast, the OD scores for p62 immunostaining in the 3-MA group were significantly higher at both 6 and 12 h time points (*p* = 0.0018 and *p* = 0.0194, respectively). Representative pictures for immunostaining for Beclin-1 and p62 are available as Supplementary Material (S2.1). Western blot analysis indicated that autophagy was enhanced in the group treated with rapamycin, as evidenced by significantly increased LC3II expression (*p* < 0.0001) and significantly increased Beclin-1 expression (*p* < 0.0001) when compared to the preservation control. By contrast, autophagy was inhibited in the group of animals treated with 3-methyladenine. We noted significant LC3II (*p* < 0.0001) and Beclin-1 (*p* = 0.0073) decreases compared to the preservation control. (Figure 4). Taken together, these results confirm that rapamycin enhances autophagy activity in the intestinal grafts, while 3-MA inhibits autophagy.

#### 2.3.2. Effects on Apoptosis

**Figure 5:**
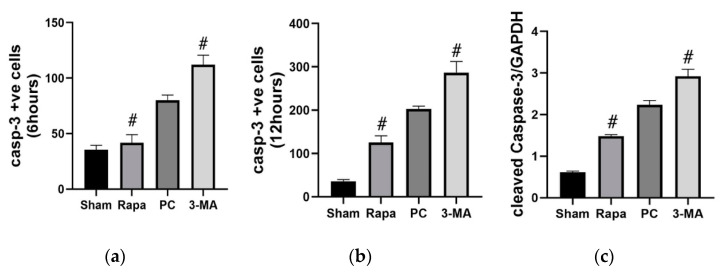
Changes in apoptosis after autophagy regulation. Panels (**a**,**b**) represent quantitative apoptotic cell counts for positively stained caspase-3 cells at 6 and 12 h, respectively. Panel (**c**) shows changes in expression of cleaved caspase-3 after 12 h of preservation. Data are mean ± SEM, n = 6. # *p* ≤ 0.05 versus PC. Representative electrophoresis pattern for cleaved caspase-3 is available as Supplementary Material (S3).

The consequence of promoting or inhibiting autophagy on apoptosis was evaluated at different time points during the preservation period (0, 6, 12 h). Immunostaining for active caspase 3 showed that at the onset of preservation (0 h), there was no significant difference between the Rapa and 3-MA groups versus the PC group (*p* = 0.6501 and *p* = 0.8278, respectively). At 6 h of preservation, there were significantly more positive-stained caspase cells in the 3-MA group than the PC group (*p* = 0.0060), while in the Rapa group, there were significantly fewer positive cells present (*p* = 0.0012) (Figure 5a). After 12 h, the 3-MA group still had a significantly higher amount of positively stained apoptotic cells (*p* = 0.0092) compared to the preservation control, while in the Rapa group, we observed fewer apoptotic cells (*p* = 0.0138) (Figure 5b). Representative pictures for immunostaining for cleaved caspase-3 are available as Supplementary Material (S3). Consistently, at the end of the 12 h long preservation period, the expression of cleaved caspase-3 in Western blot analysis also significantly increased in the 3-MA group (*p* = 0.0183) and decreased in the Rapa group (*p* < 0.0001) when juxtaposed with the PC group (Figure 5c). Altogether, these results suggest that enhancing autophagy attenuates apoptotic damage while inhibiting this autophagy process accelerates apoptotic injury.

#### 2.3.3. Effects on Intestinal Mucosa

To evaluate the role of autophagy on preservation-induced intestinal mucosa damage, we quantified Park/Chiu scores, mucosal thickness, crypt depth, and goblet cell density after 0, 6, and 12 h of preservation. Mucosal examination was unremarkable between the treated groups (Rapa and 3-MA) and the control group (PC) at the onset of preservation. Injury scores, mucosal thickness, crypt depth, and goblet cell density showed no significant difference between the groups. 

**Figure 6:**
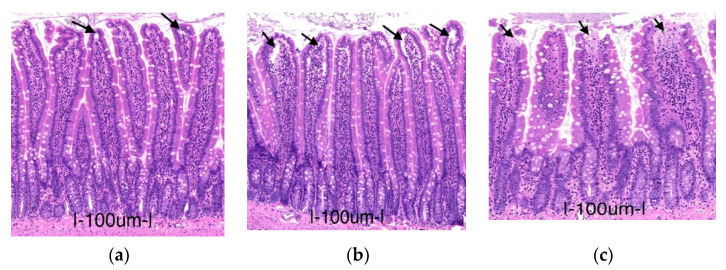
Representative pictures for mucosa changes after 6 h of preservation. Panel (**a**) represents the Rapa group. The arrows here point to subepithelial bleb at the tip of the villus, which corresponds to a Park/Chiu score of 1. Panel (**b**) represents the PC group. The arrows here point to more extended subepithelial spaces in the upper half of the villus (Park/Chiu score = 2). Panel (**c**) represents the 3-MA group. The arrows here show regions of epithelial breakdown and the denudation of villi (Park/Chiu score = 3).

After 6 h of preservation, however, the 3-MA group showed higher injury scores, characterized by areas of epithelial breakdown and some denuded villi compared to the PC group (*p* = 0.0002), while the Rapa group displayed only minimal injury in comparison, with some regions containing subepithelial blebbing at the tip of the villus (*p* = 0.0307; Figure 6). Similarly, mucosal thickness and crypt depth decreased significantly in the 3-MA group (*p* < 0.0001 and *p* = 0.00339, respectively). In the Rapa group, the values of mucosal thickness were significantly superior to the PC group (*p* = 0.0049), while the values for crypt depth were higher but not significant enough in comparison (*p* = 0.7183). Goblet cell count was significantly higher in the Rapa group compared to the PC group (*p* = 0.0104). In the 3-MA group, the number of goblet cells reduced significantly (*p* = 0.0418). 

At the end of the preservation period (12 h), the Rapa group still displayed significantly lesser tissue injury (*p* < 0.0001), superior mucosal thickness (*p* < 0.0001), and crypt depth (*p* = 0.0084) compared to the control. Similarly, the goblet cell count was significantly higher (*p* < 0.0001)). In contrast, the 3-MA group had worse injury scores, characterized by more regions of crypt damage compared to the control (*p* = 0.0050). Crypt depth was also significantly reduced (*p* = 0.0481), while the mucosal thickness was reduced but not significantly (*p* = 0.1577). Furthermore, the number of goblet cells was less in the 3-MA group (*p* = 0.2273). Picture representation after 12 h of preservation is available as Supplementary Material (S4.1). These results from the intestinal mucosa collectively suggest that enhancing autophagy attenuates preservation-induced mucosal changes while inhibiting this process proves detrimental for the graft mucosa. 

#### 2.3.4. Effects on LDH and Lactate

To quantify the effect of autophagy on the cellular markers of preservation injury, LDH and lactate release were evaluated after 6 and 12 h of storage. The findings show that the extent of preservation-induced release of LDH and lactate was attenuated in the Rapa group and worsened in the 3-MA group. After 6 h of preservation, there was less release of LDH and lactate into the preservation solution in the Rapa group compared to the PC group (*p* = 0.0015 and *p* = 0.0194, respectively). In contrast, in the 3-MA group, there was significantly more LDH and lactate in the preservation solution (*p* < 0.0001 and *p* = 0.0002, respectively). Similarly, after 12 h of preservation, increased concentrations of LDH and lactate were measured from the preservation fluid in the 3-MA group when juxtaposed with the PC group (*p* = 0.0221 and *p* = 0.0090, respectively). In the Rapa group, both LDH and lactate were significantly less concentrated (*p* < 0.0001). These results indicate that rapamycin-enhanced autophagy attenuates while inhibiting autophagy exacerbates cellular injury during intestinal preservation. Data for changes in LDH and lactate are available as Supplementary Material (S4.2).

#### 2.3.5. Reperfusion Injury

To further confirm the role of autophagy on the preservation-induced ischemia–reperfusion cascade of the intestine, at the end of the 12 h storage period, we re-perfused the grafts briefly. We compared reperfusion injury between the treated and non-treated group by quantifying the histology (Park/Chiu) scores and the release of LDH upon perfusion. The amount of LDH was measured at the end of the first minute and after the 60-min-long perfusion. Compared to the PC group, the grafts with enhanced autophagy (Rapa group) had significantly less mucosal damage (*p* < 0.0001). In contrast, the 3-MA group exhibited worse average injury scores but not significantly (*p* = 0.1119). Similarly, the amount of LDH released in the first minute and 60th minute was significantly less in the Rapa group (*p* = 0.0125 and *p* = 0.0005, respectively) compared to the PC group, while in the 3-MA group, LDH was significantly increased at both times (*p* = 0.0233 and *p* = 0.0009, respectively). These results illustrate that enhancing autophagy limits the extent of reperfusion injury while inhibiting autophagy aggravates this injury. Data for changes after reperfusion are available as Supplementary Material (S5).

#### 2.3.6. The Protective Effects of Rapa-Enhanced Autophagy Are Attenuated by 3-MA

Although rapamycin is well known as a pro-autophagic drug, some other effects, independent of autophagy, have been described in the literature [32]. Hence, we determined whether the effects observed, using rapamycin as an inducer of autophagy, were truly dependent on the autophagic pathway. We compared tissue changes, biochemical changes, and apoptotic changes at the end of the preservation period (12 h) in the Rapa group with a drug control (DC) group (animals here received both 3-methyladenine and rapamycin at the same time). The finding showed that the beneficial effects of administering rapamycin on preservation injuries were indeed due to its effect on autophagy, as evidenced by the Park/Chiu injury scores, which were significantly higher in the DC group compared to the Rapa group (*p* < 0.0001). Similarly, there were also significantly higher concentrations of LDH (*p* < 0.0001) and lactate (*p* = 0.0120) in the DC group than in the Rapa group. Picture representation for mucosal changes and changes in LDH and lactate for the DC group can be found in the Supplementary Material (S4). With regards to apoptosis, the protective role of rapamycin was still due to its pro-autophagic effect. Immunostaining for cleaved caspase-3 revealed more positive cells in the DC group than in the Rapa group (*p* = 0.0051). Similarly, the blot analysis for cleaved caspase 3 showed a significant increase in the DC group in comparison to the Rapa group (*p* < 0.0001). Representative immunostaining and immunoblots for cleaved caspase-3 for the DC group are available in the supplementary material (S5).

## 3. Discussion

Mucosal damage is the hallmark of tissue injury associated with cold preservation of the intestine. Whatever mucosal injury is present at the end of the preservation period will get worse during reperfusion. Epithelial ulceration, barrier disruption, and increased permeability of the intestinal mucosa may occur and affect clinical outcomes negatively [1,7]. Evaluating the extent of mucosal damage is, therefore, commonly employed to ascertain the integrity of intestinal grafts [1,7]. Our first study examined mucosal changes during cold preservation. We accessed mucosal damage by quantifying changes using the Park/Chiu ischemic injury score system [8], total mucosal thickness, villus depth, and goblet cell counts at different time points. The Park/Chiu scoring system can microscopically describe the progression of mucosal injury during ischemia, while the decrease in mucosal thickness and villus depth indicates atrophic changes. More so, the abundance of mucin-containing goblet cells is an important indicator of the reparative ability of the intestinal mucosa [30]. Our results show that with increasing storage time, the average Park/Chiu scores increased while mucosal thickness, crypt depth, and goblet cell count decreased. At the end of our longest storage time (12 h), we recorded the most apparent mucosal destruction. These results agree with similar studies that have correlated the extent of mucosal damage with the length of the cold ischemic time [5,33]. 

In addition to mucosal changes, we further characterized the preservation injury of the intestinal grafts by quantifying the release of two general markers of cell hypoxia and injury: lactate and lactate dehydrogenase (LDH) enzymes. Biochemical analysis of the amount of lactate and LDH released into the preservation solution demonstrated that cellular injury worsens with longer cold storage periods. This is consistent with previous studies that have used similar markers [9,31]. Taken together, our results from mucosal and biochemical analyses confirm that lengthening the cold ischemic time worsens preservation injury in small bowel grafts. 

The background of ischemic injury is dominated by several metabolic events, including oxidative and inflammatory stress, apoptosis, necrosis, and autophagic responses [22]. Over the past few years, interest in determining the role of autophagy in both warm and cold ischemic events has increased [14,18,28,34]. Autophagy, which has a basal expression level in a variety of cells, is necessary for functional homeostasis. Under conditions of starvation, hypoxemia, and energy depletion, this pathway may be upregulated. The autophagic pathway can then target damaged or redundant cytoplasmic organelles for degradation. The breakdown products of these organelles may serve as important basic metabolites for cellular reuse [11,13,21,35].

In our first experiment, we also focused on whether rat intestinal autophagy can be activated after cold preservation. Our results revealed that autophagic markers LC3II and Beclin-1 proteins showed significant upregulation after 3 and 6 h of preservation compared to the control. Accordingly, using electron microscopy, we could visualize more autophagosomes after 6 h of preservation compared to the control. This seems to confirm the upregulation of the autophagic process during the cold preservation of intestinal grafts. These results agree with other studies that show an increase in autophagy activity during the cold storage of different organs [17,26]. The autophagic pathway is, however, not without constraint. Extended states of starvation will eventually lead to a decline due to enzyme-limiting processes [35]. Accordingly, in our results, after the peak at 3 h of preservation, we noted a steady decrease in LC3II and Beclin-1 proteins. Both LC3II and Beclin-1 proteins show a significant decrease after 12 h of preservation compared to the operated control. We offer two possible explanations for this: First, it is likely that the baseline used for our study (0 h, operated control group) is not the true basal expression of autophagy in intestinal cells. Since our experimental animals were starved for 24 h as part of the protocol and starvation is a strong moderator of autophagy, we suspect that the autophagic process was already upregulated in the intestine before we retrieved the organ. Accordingly, the baseline we adopted is a good one for monitoring changes in autophagy within a limit but should be interpreted cautiously. Secondly, in some instances, prolonged periods of ischemia (starvation) impair autophagy. The consumption and depletion of essential components for autophagy after sustained starvation and the inhibition of key regulators such as Beclin-1 by caspases are said to be behind this phenomenon [25,36]. The exact reason for this occurrence in our results was not investigated further. These time-based differences in the expression of autophagic proteins do, however, suggest a role for autophagy in cold intestinal preservation.

The role of autophagy during warm or cold ischemia varies amongst the different organs [14,19,20,26]. Therefore, in our second experiment series, we investigated the influence of autophagy on the cold preservation ischemia of small bowel grafts. From our results, inhibiting the upregulation of autophagy with 3-methyladenine resulted in a more pronounced preservation injury. The effects of this inhibition were already prominent at 6 h of preservation, lasting until after 12 h of preservation. This suggests that inhibition of the cold-ischemia-induced autophagy accelerates the damage to small bowel grafts. In contrast, by enhancing intestinal autophagy using rapamycin, preservation injury of the intestine was attenuated after 6 h and at the end of the 12-h-long preservation period. Additionally, when the autophagy inhibitor, 3-methyladenine, was added to animals that also received rapamycin, the protective effects of enhancing autophagy were diminished. Collectively, our results indicate that autophagy is protective and that enhancing this pathway will reduce the preservation injury of small bowel grafts.

During severe cold ischemia–reperfusion injury of the intestine, the breakdown of the mucosal barrier function enables bacterial translocation and subsequent septic events. A correlation has previously been established between the rate of this occurrence and the length of the cold ischemic time [23,37]. In view of this, intestinal grafts preserved for extended periods (over 9–10 h) are generally not utilized [10,23]. Ways to prolong the preservation time for intestinal grafts are continually being proposed [3]. In our second study, at the end of the extended preservation period (12 h), we carried out short-term reperfusion. Our results revealed that reperfusion injury was significantly less in the grafts where autophagy was enhanced. Those grafts displayed better mucosal morphology and less release of LDH into the perfusate. 

In terms of mucosal damage during ischemia–reperfusion injury, our results contrast those obtained by Li Baochuan et al. They monitored autophagy in the warm ischemia–reperfusion injury of the intestine and concluded that enhanced autophagy was detrimental for the mucosa layer [14]. We measured changes in autophagy after cold storage, as well as mucosa damage after preservation and reperfusion, and we propose a beneficial role. Although the eventual outcome of warm and cold ischemia–reperfusions are similar, their mechanisms develop differently [19]. During cold storage, the preserved organ is entirely cut off from the ‘milieu’ of tissue perfusion and depends solely on its intrinsic adaptation. In an ongoing warm ischemia–reperfusion injury, however, the possible influence of collateral blood supply on metabolism cannot be ruled out. Furthermore, differences in autophagic adaptation within similar pathologies have been described in the literature [19,38]. Taken together, the need for continuous studies into autophagy is further emphasized.

The protective effects of autophagy have been ascribed, at least in part, to its influence on the apoptotic death pathway [24]. The relationship between autophagy and apoptosis is rather dynamic and depends on the nature of the stimuli [24,25]. In a cell undergoing stress, both pathways can be activated. Autophagy is generally more beneficial and serves to promote cell survival. It can also inhibit apoptosis propagation. However, in some cases, autophagy may induce cell death as well, known as autophagic cell death [25]. Caspase-3 activation and enterocyte apoptosis have been previously reported as facilitators of intestinal damage during cold storage [6,30]. By manipulating autophagy, we determined its effect on cold preservation-induced apoptosis. From the results, enhancing autophagy reduced Caspase-3 activity whilst inhibiting autophagy had the opposite effect. Furthermore, the antiapoptotic role of rapamycin-enhanced autophagy was significantly reduced when 3-methyladenine was also administered in the same animals. Accordingly, we can infer that enhancing autophagy has a positive effect on the cold preservation-induced apoptosis of intestinal mucosa.

In summary, our results point to the involvement and possible role of autophagy in cold preservation injury of the intestine. We observed that enhanced autophagy was protective against cold ischemia and reperfusion damage of small bowel grafts. However, in this experiment, we did not consider all the different cell populations (such as Paneth and immune–modulatory-type cells) present in the intestine. Further studies will be needed to dissect the influence of autophagy on these cells during cold preservation. Nonetheless, the findings of this study provide new insights for researchers in this field. 

## 4. Materials and Methods

### 4.1. Experimental Animals

Healthy male Wistar rats (n = 60), weighing between 250–300 g, were used for this study. They were housed under standard conditions and fed rat chow and water ad libitum. Food was withdrawn 24 h prior to the experiment. Animals were anesthetized with an intraperitoneal (i.p) mixture of ketamine hydrochloride (0.075 mg/g of body weight) and diazepam (0.075 mg/g of body weight). All procedures were performed in accordance with ethical guidelines (BA02/2000-02/2021) to minimize the pain and suffering of the animals.

### 4.2. Intestinal Procurement and Grouping

After median laparotomy, the intestine was retrogradely perfused via the aorta at 6 mL/min with ice-cold IGL-1 solution (Institute George Lopez) for 2 min. The portal vein was cut to facilitate venous venting. At the end of the perfusion, small bowel grafts were resected from the ligament of Treitz and stored in the same solution at 4 °C.

In the first series of the experiment, the rats were randomly divided into an operated control group (0 h; n = 6) and a preservation group. The preservation group was subdivided into 4 groups—3, 6, 9, and 12 h groups (n = 6 for each group)—based on the duration of cold storage. Intestinal samples were collected after the resection (0 h; control), and at the end of the preservation periods for Western blot (wb), histology, immunohistochemistry (IHC), and biochemical and transmission electron microscopy (TEM) analyses. 

In the second series of the experiment, rats were randomly divided into 5 groups (n = 6 for each group): the sham-operated group (Sham), the preservation control group (PC), the preservation group treated with rapamycin (rapamycin group), the preservation group treated with 3-methyladenine (3-MA group), and the drug control group (DC). A promoter of autophagy, rapamycin (Hb2779 Hellobio; 2 mg/kg dissolved in 1 mL dimethyl sulfoxide solution (DMSO), i.p), was injected into rats belonging to the rapamycin group, 30 min before organ retrieval; an inhibitor of autophagy, 3-MA (Hb2267 Hellobio; 2 mg/kg dissolved in 1 mL ddH20, i.p), was injected into the 3-MA group, 30 min before organ retrieval [14]. The drug control group received the same dose of both rapamycin and 3-methyladenine at the same time. The sham-operated group and the preservation control groups received the same volume of the solvents (ddH20 and DMSO) used. The total time of preservation was 12 h. After cold storage, grafts were perfused using oxygenated Krebs Henslet buffer solution (KHBS) for 60 min according to an ex-vivo method described previously [39]. Intestinal samples and preservation and perfusion fluids were taken at various time points for further analysis.

### 4.3. Histology (Hematoxylin–Eosin)

Tissues were fixed in 10% neutral buffered formalin and embedded in paraffin. They were cut in 3-micrometer-thick sections and stained with hematoxylin and eosin. The slides were digitized with a Mirax scanner, and photographs were taken with CaseViewer 2.4 software (3DHISTECH Ltd. Budapest, Hungary). Intestinal mucosa damage was evaluated blindly by two individuals. The degree of injury was determined using the Park/Chiu system described by Park et al. [8]. A minimum of three fields randomly selected from four quadrants of each intestinal sample was evaluated.

Morphometric analysis of total mucosa thickness and villous depth was analyzed using CaseViewer 2.4 software (3DHISTECH Ltd. Budapest, Hungary). Total mucosa thickness was assessed by measuring the distance between the villus tip to the lamina-muscularis mucosae in at least four axially oriented villi in four quadrants. Crypt depth was determined in at least a total of five axially oriented, open, non-destroyed crypts from three quadrants. 

### 4.4. Goblet Cell Count

To evaluate the amount of mucus containing goblet cells in the mucosal layer, tissues were fixed in 10% neutral buffered formalin, embedded in paraffin, cut into 3-micrometer-thick sections with a rotational microtome and mounted on coated glass microscope slides. After deparaffinization and rehydration, the samples were incubated in 1% periodic acid for 20 min, followed by a 0.5 min rinse in distilled water. The samples were stained with Schiff reagent for 20 min, differentiated in Schiff-rinsing solution for 2 min, and immersed in tap water for 5 min to further evolve their color. Slides were then incubated in Meyer’s hematoxylin for 10 min, and bluing was performed with tap water for 5 min. The samples were dehydrated in alcohol, cleared in xylene, and mounted with permanent mounting medium. The amount of blue/purple-stained goblet cells was evaluated by manually counting the number of visible cells in at least three high-power fields (hpfs) selected randomly from four different quadrants using Caseviewer 2.4 software (3DHISTECH Ltd., Budapest, Hungary).

### 4.5. Immunohistochemistry (IHC)

Intestinal tissues, fixed in 10% neutral buffered formalin and embedded in paraffin, were cut in serial 3-micrometer-thick sections. After deparaffinization and rehydration, samples were pretreated with the heat-induced epitope retrieval method in 1 mM (pH = 6.0) citrate buffer (Histopathology Ltd.Pécs, Hungary) in a microwave oven for 15 min at 750 W. After cooling at room temperature, the tissues were washed in TRIS buffered saline solution (TBS) (pH = 7.6). For immunohistochemistry, samples were incubated in Beclin-1 antibody (Cat. Nr. bs-1353R, Bioss Antibodies Inc., 1:2000, 1 h at room temperature), p62/sqstmi (Cat. Nr. p0067. Sigma-Aldrich Ltd., USA 1:2000, 1 h at room temperature), and cleaved caspase-3 (Asp175, Cat. Nr. 9661, Cell Signaling Technology, Inc., USA 1:100, 1 h at room temperature). The sections were washed in TBS and incubated with a HISTOLS-R anti-rabbit HRP-labeled detection system (Cat. Nr. 30011.R500, Histopathology Ltd., 30 min at room temperature). After washing in TBS, the reaction was developed with a HISTOLS Resistant AEC Chromogen/Substrate System (Cat. Nr. 30015, Histopathology Ltd.) while controlling the intensity of the staining under a microscope. Sections were counterstained with hematoxylin solution, and bluing was performed with tap water. Samples were dehydrated in alcohol, cleared in xylene, and mounted with permanent mounting medium. Slides were digitized with a Mirax scanner, and photographs were taken with CaseViewer 2.4 software (3DHISTECH Ltd.Budapest, Hungary). 

Analysis of Beclin-1 and p62/sqstmi stains was performed with the help of the IHC profiler plug-in of Image J software, and the optical density (OD) was scored according to the method described previously [40].

Analysis of the cleaved-caspase-3-stained cells was achieved by manually counting the visibly stained apoptotic cells in at least four high-power fields (hpfs) randomly selected from four different quadrants using CaseViewer 2.4 software.

### 4.6. Western Blot (wb)

Intestinal tissue samples were frozen in liquid nitrogen and then manually pulverized in a mortar and dissolved in ice-cold lysis buffer (containing 50 mM Tris, pH 7.4, 150 mM NaCl, 1 mm EGTA, 1 mM Na_3_VO_4_, 100 mM NaF, 5 μM ZnCl_2_, 10% glycerol, and 1% Triton X-100 plus 10 μg/mL of the protease inhibitor aprotinin). Lysates were subjected to centrifugation at 40,000× *g* at 4 °C for 30 min, then the protein concentration of the supernatants was determined using Protein Assay Dye Reagent Concentration (Bio-Rad Gmbh München, Germany) and light absorption measurement at 595 nm. Samples containing 30 ug of denatured total protein were prepared and loaded onto 10% polyacrylamide gels. Proteins separated based on size were electro-blotted for half an hour onto PVDF membranes using the Trans-Blot Turbo semi-dry system (Bio-Rad), then blocked in 3% BSA dissolved in Tris-buffered saline containing 0.2% Tween 20. This was followed by the probing of the membranes with the primary antibodies (Beclin-1, LC3 (Cat. Nr. 2775 and Cat. Nr.4108 Cell Signaling Technology), and cleaved caspase-3), diluted 1:1000 in the blocking solution, at 4 °C overnight. The binding of the antibodies to the membrane was detected by a secondary anti-rabbit IgG conjugated to horseradish peroxidase (Santa Cruz Biotechnology. Texas, USA), diluted 1:10,000. The enhanced chemiluminescent signal was visualized using a Gbox gel documentation system (Syngene, UK). All membranes were then stripped from the antibodies and detected again, as above, for possible loading differences using a primary antibody against GAPDH (Cell Signaling Technology) at a dilution rate of 1:3000. Analysis of band densities was performed using Image J software. Each of the densities was further quantified in relation to GAPDH. 

### 4.7. TEM

The intestines were cut into large blocks of approximately 1 mm^3^ and fixed overnight at 4 °C in 4% paraformaldehyde with 2.5% glutaraldehyde in phosphate buffer (PB). These blocks were then fixed in 1% osmium tetroxide in 0.1 M PB for 35 min after dehydration in an ascending ethanol series, with uranyl acetate (1%) included in the 70% ethanol stage to increase contrast. The blocks were transferred to propylene oxide before being placed into aluminum foil boats containing Durcupan resin (Sigma) and then embedded in gelatin capsules containing the same resin. Semithin sections were cut with a Leica ultramicrotome and mounted either on mesh or Collodion-coated (Parlodion, Electron Microscopy Sciences, Fort Washington, PA, USA) single-slot copper grids. Additional contrast was provided to these sections with uranyl acetate and lead citrate, and they were examined in a JEM-1400 flash transmission electron microscope.

### 4.8. Biochemical Analysis

At different time points during preservation and reperfusion, fluid samples were obtained and analyzed for the presence of lactate and lactate dehydrogenase enzyme. After centrifugation (10 min, room temperature, 1500 rcf), both parameters were quantified using the Cobas Integra 400 Plus Analyzer (Roche Diagnostics, GmbH, Mannheim, Germany), following the manufacturer’s instructions.

### 4.9. Statistical Analysis

For statistical evaluation, a one-way analysis of variance (ANOVA) was used, followed by adequate posthoc tests for multiple comparisons. The Kruskal–Wallis test was used for the analysis of histological (Chiu) scores. Comparing changes within a group was performed using the paired *t*-test. All data are represented as the mean ± SEM. The difference was considered statistically significant when the *p*-value was less than 0.05.

## Figures and Tables

**Table 1:** Mucosal injury scores after cold preservation.

Groups	0 h	3 h	6 h	9 h	12 h
**Post-preservation**	0.16 ± 0.04	1.13 ± 0.09 *	2.21 ± 0.12 *	2.86 ± 0.10 *	4.27 ± 0.10 *

Data are mean ± SEM, n = 6. * *p* < 0.0001 versus the 0 h group (operated control).

**Table 2:** Biochemical analysis of preservation fluid.

Groups	0 h	6 h	12 h
**LDH (IU/L)**	27.44 ± 2.17	349.40 ± 15.86 *	716.30 ± 34.02 *
**Lactate (mmol/L)**	0.07 ± 0.01	0.68 ± 0.06 *	1.55 ± 0.06 *

Data are mean ± SEM, n = 6. * *p* < 0.0001 versus 0 h.

## Data Availability

Data are available in the text and upon request from the corresponding author.

## Supplementary Materials

The following are available online at https://www.mdpi.com/article/10.3390/metabo11060396/s1. Figure S1: Changes in intestinal mucosa after varying periods of preservation. Figure S2: Impact of autophagy regulation on the expression of LC3II, Beclin-1, and caspase-3 proteins. Figure S3: Influence of autophagy regulation on cold ischemia and reperfusion mucosa damage.
