# Supplementary material for: Characterizing Autophagy in the Cold Ischemic Injury of Small Bowel Grafts: Evidence from Rat Jejunum
Source: Metabolites. 2021 Jun 17;11(6):396. doi: 10.3390/metabo11060396 (PMC8234201; doi:10.3390/metabo11060396)
Supplement: Supplementary file 1 [file metabolites-11-00396-s001.zip › metabolites-1188644-supplementary.pdf]

Characterizing Autophagy in the Cold Ischemic Injury of Small Bowel Grafts: Evidence from Rat Jejunum.

Ibitamuno Caleb[1]*, Luca Erlitz[1], Vivien Telek[1], Mónika Vecsernyés[2], György Sétáló Jr. [2], Péter Hardi[1], Ildikó Takács[1], Gábor Jancsó[1], Tibor Nagy[1].

1. Institute of Surgical Research and Techniques, University of Pécs Medical School, Pécs, Hungary. Email: caleb.ibitamuno@pte.hu
2. Institute of Medical Biology and Central Electron Microscope Laboratory, University of Pécs Medical School, Pécs, Hungary.
*Correspondence: ibical@yahoo.com

Supplementary Materials

S. 1: Transmission Electron Microscope photographs.

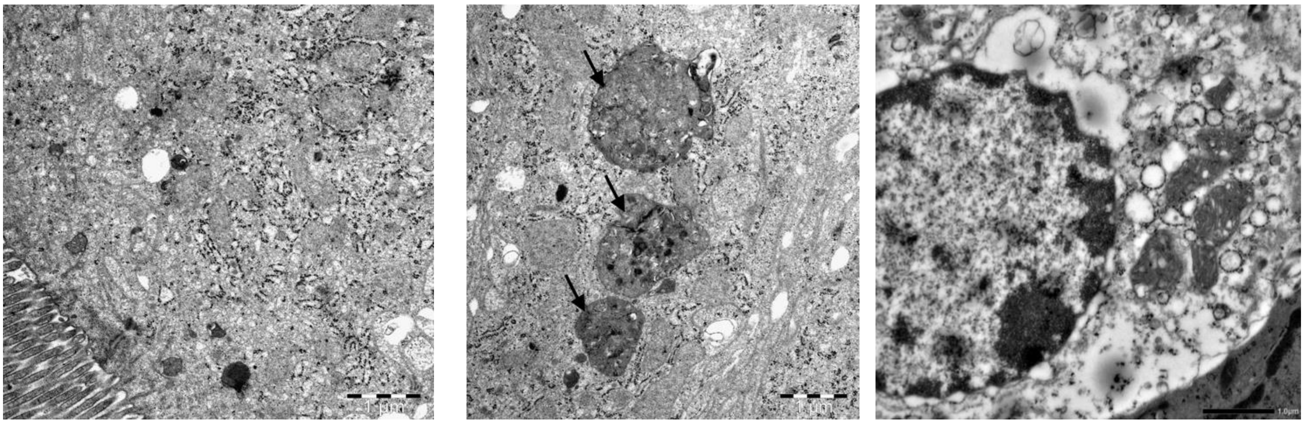

(a) (b) (c)

S. 1 – Representative TEM images from the cytoplasm of intestinal villi epithelial cells. Scale bar: 1.0μm. Panels a represents the 0hr group; Panel b represents the 6hr and Panels c represents 12hr group. In the 6hr group, the presence of autophagosomes (membrane bound structure containing cytoplasmic structures: black arrows) are clearly visible.

S. 2: Changes in autophagy after pharmacological manipulation using rapamycin and 3-methyladenine.

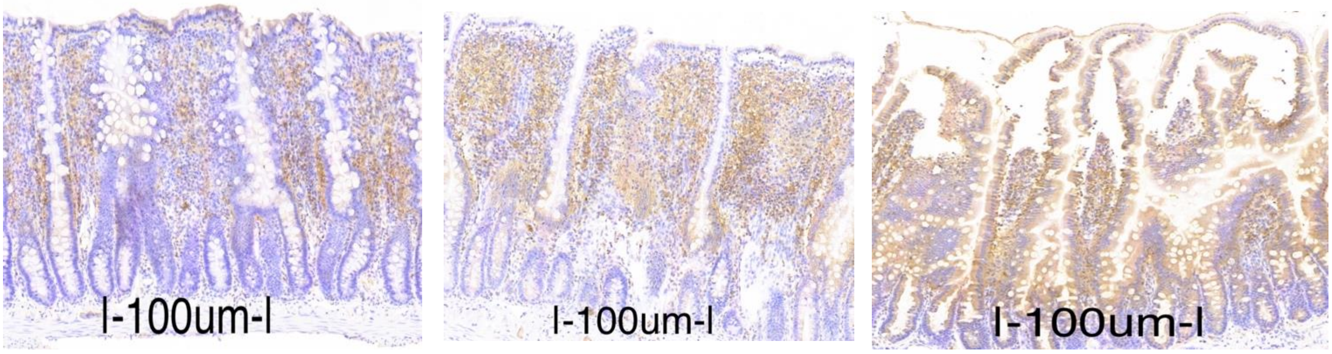

(a) **(b)** **(c)**

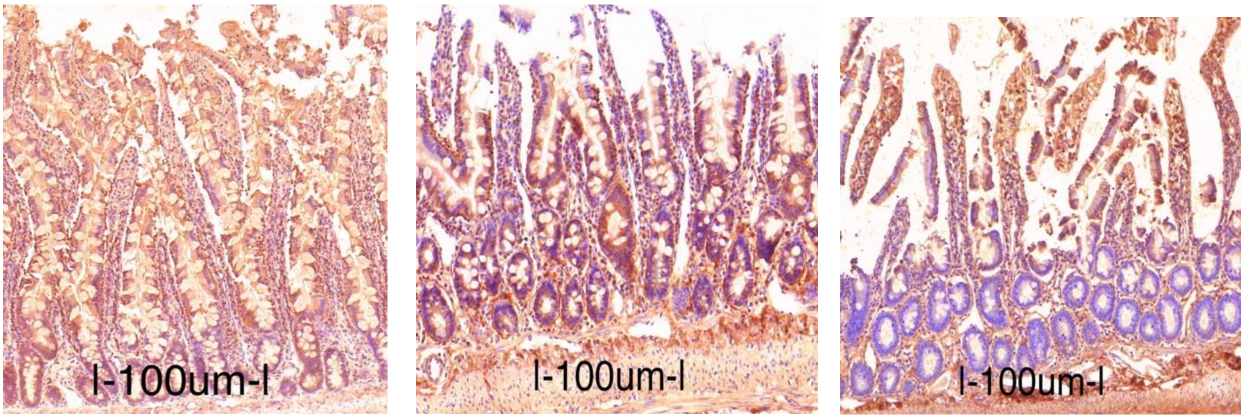

(d) **(e)** **(f)**

S. 2.1 – Immunostaining for p62 and Beclin-1. Panel a-c represent changes in the immunostaining for p62 protein after 6 hours of preservation. Panel a represents the Rapa group; Panel b represents the PC group; Panel c represents the 3-MA group. Panel d-f represent changes in immunostaining for Beclin-1 at the end of preservation (12 hours). Panel d represents the Rapa group; Panel e represents the PC group; Panel f represents the 3-MA group. Both proteins stain the cytoplasm of the intestinal epithelial cells brown. Scale bar: 100um.

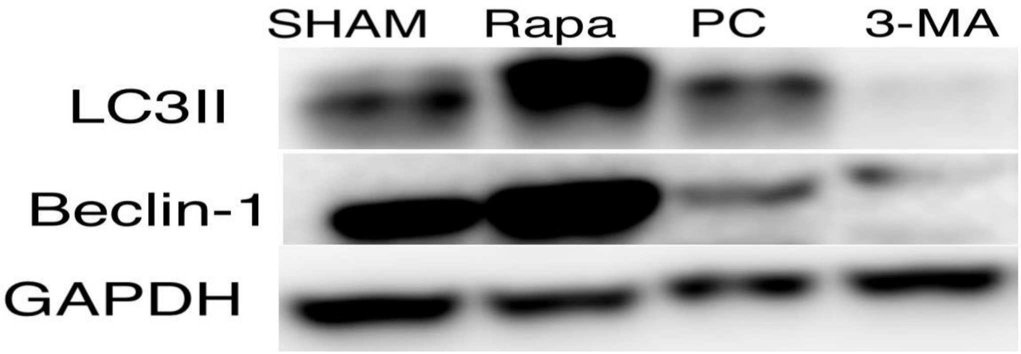

(a)

S2.2 – Panel a show the changes in electrophoresis pattern for LC3II and Beclin-1 at the end of the preservation period.

S. 3: Influence of autophagy on apoptosis

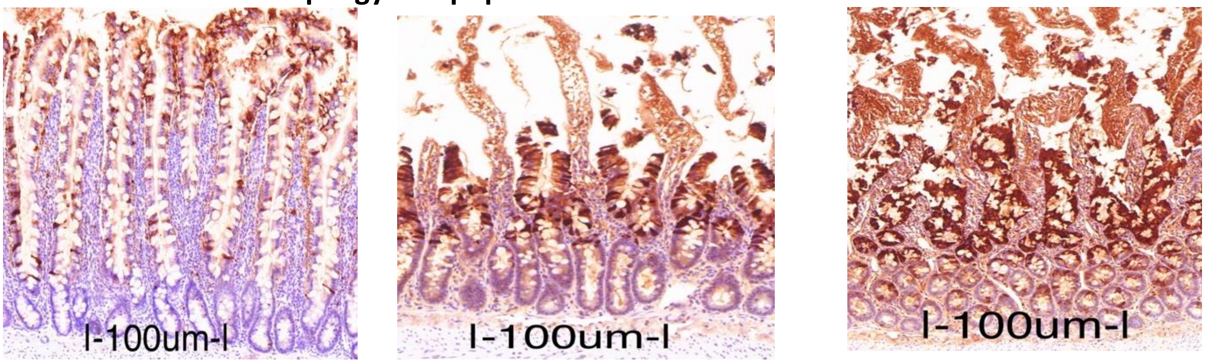

(a) **(b)** **(c)**

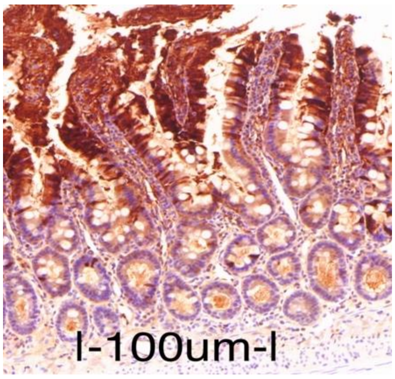

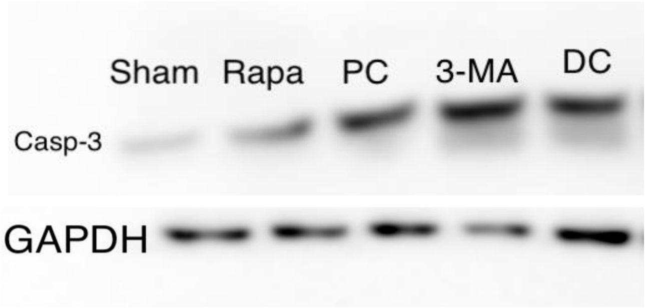

(d) **(e)**

S. 3 – Effect of autophagy on apoptosis after 12 hours of preservation. Panel a-d are picture representation of immunostaining for active caspase-3. Dark brown staining of the cytoplasm is considered a positive stain. The Rapa group is represented in Panel a showing few positive stains. Panel b represents the PC group, showing more positive cells than the Rapa group. 3-MA group is represented by Panel C, here there is a strong positivity for caspase-3. The DC group (Panel D) also shows positive cytoplasmic stain. When compared the Rapa group has the least positivity among the groups. Panel e shows the representative electrophoresis pattern for the cleaved caspase-3.

S. 4: Influence of autophagy on preservation injury

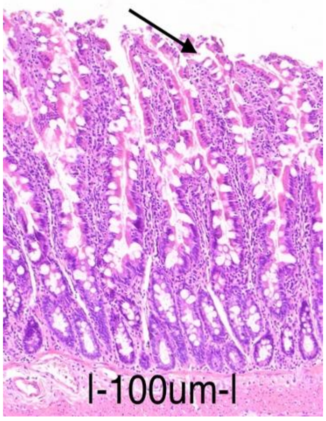

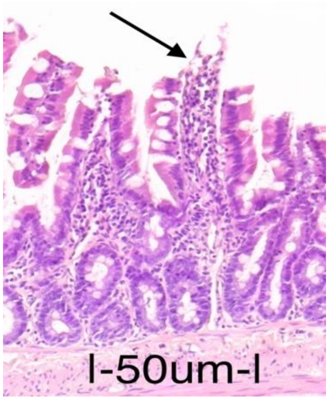

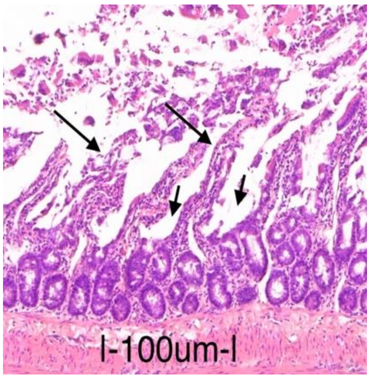

(a) **(b)** **(c)**

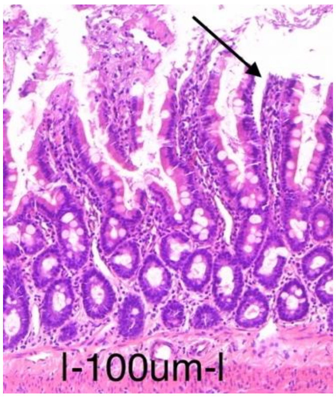

(d)

S. 4.1 – effect of autophagy on the intestinal mucosa. Panel a-d represent pictures from HE staining of the small bowel graft after 12 hours of preservation. Panel a is from the Rapa group, the arrow points to extended epithelial space at the tip of the villus (Park/Chiu score 2) which was characteristic for mucosa of this group. Panel b is from the PC group, arrow points to the characteristic incomplete denudation of villi with some loss of villous tissue (Park/Chiu score 4-5). Panel c is from the 3-MA group which shows complete villous denudation (long arrow) and some loss of villous tissue (short arrow) (Park/Chiu =5). Panel d is from the DC group, the epithelium also shows regions of villous tissue loss and incomplete denudation(arrow) (Park/Chiu score 4-5).

Groups	Sham	Rapa	PC	3-MA	DC
LDH (IU/L)	25.61±1.49	425.61±11.79#	593.30±22.68	685.40±40.32 #	558.00±20.16 ^
Lactate (mmol/l)	0.06±0.01	1.06±0.11#	1.55±0.06	1.83±0.04#	1.53±0.14^.

S. 4.2 – Table showing changes in Ldh and lactate levels at the end of the 12-hour long cold storage. Data are mean ± SEM. #P < 0.05 versus PC. ^P < 0.05 DC versus Rapa group.

S.5: Reperfusion Injury

Groups	Sham	Rapa	PC	3-MA
Park/Chiu scores	0.94±2.75	2.86±0.11#	5.04±0.11	5.76±0.12
LDH (IU/L) - 1 min	72.71±9.66	103.680±6.01#	145.20±10.38	183.30±12.64#
LDH (IU/L) - 60min	210.80±16.45	332.47±198.17#	433.23±20.46	530.20±16.53#

S.5 – Table showing changes in Park/Chiu scores and Ldh levels corresponding to the reperfusion injury. Data are mean ± SEM. #P < 0.05 versus PC. group.
